# Supplementary material for: MLe-KCNQ2: An Artificial Intelligence Model for the Prognosis of Missense KCNQ2 Gene Variants
Source: Int J Mol Sci. 2024 Mar 2;25(5):2910. doi: 10.3390/ijms25052910 (PMC10932340; doi:10.3390/ijms25052910)
Supplement: Supplementary file 1 [file ijms-25-02910-s001.zip › Description Supplemental Material.pdf]

Supplemental material description:

- AlphaFold2_KCNQ2_Prediction.pdb: PDB file containing the highest ranked KCNQ2 structure predicted by AlphaFold2.

- Supp_Alignment.xlsx: Multiple sequence alignment of KNCQ2 sequences used to calculate evolutionary conservation.

- Supp_Data_1_FINAL_DATASET_KCNQ2.xlsx: Set of variants used for comparison of different predictive models.

- Supp_Data_2_KCNQ2_Performance_Tools.xlsx: Performance, measured in sensitivity, specificity and AUC-ROC, of all predictive models for the set of variants in the Supp_Data_1_FINAL_DATASET_KCNQ2.xlsx file

- Supp_Data_3_KCNQ2_All_Predictions.xlsx: Predictions made by our model for all variants in our dataset (both train and test sets).

- Supp_Data_4_All_Possible_Predictions_no_codon.xlsx: Predictions made by our model for all possible amino acid changes in the KCNQ2 amino acid sequence.

- Supp_Data_5_All_Possible_Predictions.csv: Predictions made by our model for all possible nucleotide changes in the KCNQ2 gene.

- Supp_Feature_Description.docx: Detailed description and numerical values (when appropriate) of all the features employed for model development.

- AUC_ROC_Scores.xlsx: AUC-ROC scores computed for the models compared in this study.
