# Supplementary material for: MLe-KCNQ2: An Artificial Intelligence Model for the Prognosis of Missense KCNQ2 Gene Variants
Source: Int J Mol Sci. 2024 Mar 2;25(5):2910. doi: 10.3390/ijms25052910 (PMC10932340; doi:10.3390/ijms25052910)
Supplement: Supplementary file 1 [file ijms-25-02910-s001.zip › Supp_Feature_Description.pdf]

SUPPLEMENTARY MATERIAL

Physico-chemical changes features

The physiological pH value of nerve cells is 7.4 (Jalalvand et al., 2016). The basic physico-chemical properties of the amino acids used to generate these descriptors are listed in Table S1.

Table S1. Physico-chemical properties of the amino acids at pH = 7.4

Amino acid	Charge	Polarity	Aromaticity	Size (Da)	Standardized volume (%)	Medium solvent accessibility (%)	Hydrophobicity
(Ser) S	neutral	polar	non aromatic	105.09	18.1	40.5	-0.8
(Thr) T	neutral	polar	non aromatic	119.12	34	35.3	-0.7
(Gln) Q	neutral	polar	non aromatic	146.15	51.3	43.6	-3.5
(Asn) N	neutral	polar	non aromatic	132.12	35.4	46.1	-3.5
(Tyr) Y	neutral	non polar	aromatic	181.19	78.5	30.1	-1.3
(Cys) C	neutral	polar	non aromatic	121.16	28	7.4	2.5
(Gly) G	neutral	polar	non aromatic	75.07	0	54	-0.4
(Ala) A	neutral	non polar	non aromatic	89.09	15.9	37.4	1.8
(Val) V	neutral	non polar	non aromatic	117.15	47.7	19.6	4.2
(Leu) L	neutral	non polar	non aromatic	131.17	63.6	10.1	3.8
(Ile) I	neutral	non polar	non aromatic	131.17	63.6	7.5	4.5
(Met) M	neutral	non polar	non aromatic	149.21	62.8	3.9	1.9
(Pro) P	neutral	non polar	non aromatic	115.13	41	66.2	-1.6
(Phe) F	neutral	non polar	aromatic	165.19	77.2	5.5	2.8
(Trp) W	neutral	non polar	aromatic	204.23	100	13.8	-0.9
(Asp) D	negative	polar	non aromatic	133.1	31.3	45	-3.5
(Glu) E	negative	polar	non aromatic	147.13	47.2	48.6	-3.5
(Lys) K	positive	polar	non aromatic	146.19	68	54.3	-3.9
(Arg) R	positive	polar	non aromatic	174.2	70.8	50.1	-4.5
(His) H	positive	polar	non aromatic	155.16	49.2	28.1	-3.2

1. Quantitative changes (*d_size*, *d_hf*, *d_vol* and *d_msa*)

Quantitative changes (e.g., changes in size (*d_size*), hydrophobicity (*d_hf*), volume (*d_vol*), mean solvent accessibility (*d_msa*)) that occur between the original amino acid and the one resulting from the substitution. These descriptors were calculated by subtracting the value of the original amino acid to the new one. The hydrophobicity of the amino acids was obtained from from (Kyte and Doolittle, 1982) and both volume and average solvent accessibility from (Bogardt, Jr. et al., 1980).

2. Qualitative changes (*d_charge*, *d_pol* and *d_aro*)

Qualitative changes (e.g., changes in charge (*d_charge*), polarity (*d_pol*) and aromaticity (*d_aro*)) that occur between the original amino acid and the one resulting from the substitution. To determine the charge change of the amino acids, they were first classified according to their charge and then a unique code was generated for each of the possible charge changes. E.g.: If the initial amino acid has positive charge (pos) and the resultant of the mutation has negative charge (neg), the change is set as pos_to_neg; if the initial amino acid has neutral charge (neu) and the resultant of the mutation has

positive charge (pos), the change is set as neu_to_pos, etc. The same procedure is performed for changes in polarity and in aromaticity.

Biological-evolutive features

1. Original and mutated residues (*initial_aa* and *final_aa*)

These data were extracted directly from the nomenclature of the collected variants. E.g.: in the case of "L101H": "L" corresponds to the original amino acid and "H" to the amino acid resulting from the substitution (final).

2. Functional and topological affected domains (*functional_domain* and *topological_domain*)

The main topological and functional domains of K_V7.2 were obtained: (1) directly from information available from Uniprot (https://www.uniprot.org/) (Uniprot ID: O43526) (2022), (2) from the PDB (PDB DOI: https://www.rcsb.org/structure/7CR3) (https://www.rcsb.org/) (Berman et al., 2003), (3) from information currently known in the laboratory and (4) from other bibliographic sources (Tables S2 y S3).

Table S2. Main topological domains of the K_V7.2 channel.

Topological domain	Initial residue	Final residue	Reference
S1	A92	F112	Uniprot (O43526)
S2	E123	V143	Uniprot (O43526)
S3	P167	S187	Uniprot (O43526)
S4	A196	W218	Uniprot (O43526)
S5	L232	L252	Uniprot (O43526)
Pore	A265	P285	Uniprot (O43526)
S6	L292	L312	Uniprot (O43526)
hA	R332	N350	RSCB PDB (7CR3)
hTW	H357	T366	RSCB PDB (7CR3)
hB	G535	L559	RSCB PDB (7CR3)
hC	D563	G594	RSCB PDB (7CR3)
hD	R622	R647	Bernardo-Seisdedos (2015)

Table S3. Main functional domains of the K_V7.2 channel.

Functional domain	Initial residue	Final residue	Reference
Voltage sensor domain (S1-S4)	A92	W218	Ambrosino *et al.* (2015)
Selectivity filter	T277	D282	Uniprot (O43526)
Pore domain (S5-S6)	L232	L312	Bañales-Belaunde (2019); Yus-Nájera et al. (2002)
CaM interaction (hA-hTW-hB)	R332	L559	Ambrosino *et al.* (2015)
SID domain (hC-hD)	D563	R647	Bernardo-Seisdedos (2015)
Unknown function	*Residues not included in any of the above ranges*		

3. <u>Evolutive conservation value across species of the substituted residue (*residue_conserv*)</u>

The evolutive conservation value of each residue (residue_conserv) in the K_v7.2 channel (*KCNQ2*) was calculated by performing a Multiple Sequence Alignment (MSA) with Clustal Omega (https://www.ebi.ac.uk/Tools/msa/clustalo/) (Sievers and Higgins, 2018). A total of 62 species available in Uniprot were considered for this analysis. Species with similar sequence length for the channel were aligned, and the evolutive conservation value was calculated using the software of (Capra and Singh, 2007) available at: https://compbio.cs.princeton.edu/conservation/score.html. The conservation values obtained for each residue and the 62 species considered are shown in Data S2 and Table S4, respectively.

Table S4. Species from which *KCNQ2* sequences were obtained for the estimation of evolutive conservation value. The sequences of all species (62) range from 850 to 875 amino acids.

UniProt Code	Specie	UniProt Code	Specie
A0A6J1YBD7	*Acinonyx jubatus*	A0A6J1VM80	*Notechis scutatus*
A0A7N5JUP3	*Ailuropoda melanoleuca*	A0A7L4BX40	*Nyctiprogne leucopyga*
A0A2K5EWT1	*Aotus nancymaae*	A0A6P3FS62	*Octodon degus*
A0A6P3GU07	*Bison bison bison*	A0A2U3WMF7	*Odobenus rosmarus divergens*
G3N2R2	*Bos taurus*	A0A6J0W124	*Odocoileus virginianus texanus*
F7CY15	*Callithrix jacchus*	A0A7L1ERT0	*Oenanthe oenanthe*
A0A452E7F3	*Capra hircus*	H0X1I7	*Otolemur garnettii*
A0A286XK97	*Cavia porcellus*	A0A6P3YM10	*Ovis aries*
A0A2K5QX14	*Cebus imitator*	A0A2R9AU86	*Pan paniscus*
A0A2K5L1K5	*Cercocebus atys*	H2QKS4	*Pan troglodytes*
A0A7L3EB03	*Chaetops frenatus*	A0A6P9E1I7	*Pantherophis guttatus*
A0A6J2VKS8	*Chanos chanos*	A0A2I3NHI2	*Papio anubis*
A0A6I9KEP6	*Chrysochloris asiatica*	A0A6J0CPL8	*Peromyscus maniculatus bairdii*
A0A6J2PDX4	*Cottoperca gobio*	A0A6P5JXG8	*Phascolarctos cinereus*
A0A2Y9Q6H4	*Delphinapterus leucas*	A0A6J2MPK5	*Phyllostomus discolor*
A0A4W4F1P1	*Electrophorus electricus*	A0A2Y9T7Y1	*Physeter macrocephalus*
F6VHK7	*Equus caballus*	A0A2K6ET95	*Propithecus coquereli*
A0A1S3WCY9	*Erinaceus europaeus*	O88943	*Rattus norvegicus*
M3WAG9	*Felis catus*	A0A671F4Z0	*Rhinolophus ferrumequinum*
G3QZ98	*Gorilla gorilla gorilla*	A0A2K6P629	*Rhinopithecus roxellana*
A0A7K9DPE3	*Hemiprocne comata*	A0A7N4NU17	*Sarcophilus harrisii*
O43526	*Homo sapiens*	A0A672MMS1	*Sinocyclocheilus grahami*
A0A2D0RWX1	*Ictalurus punctatus*	A0A286ZS06	*Sus scrofa*
A0A287DBX6	*Ictidomys tridecemlineatus*	A0A6J3Q231	*Tursiops truncatus*
A0A6J0IPQ1	*Lepidothrix coronata*	A0A452SKB9	*Ursus americanus*
A0A340XT99	*Lipotes vexillifer*	A0A3Q7W3Y1	*Ursus arctos horribilis*
A0A2K6C5Y1	*Macaca nemestrina*	A0A6J3BGW9	*Vicugna pacos*
A0A1U8BXY3	*Mesocricetus auratus*	A0A4X2LHR0	*Vombatus ursinus*
F6WQP9	*Monodelphis domestica*	A0A3Q7U035	*Vulpes vulpes*
B7ZBV9	*Mus musculus*	A0A6I8QM30	*Xenopus tropicalis*
A0A341BVZ4	*Neophocaena asiaeorientalis*	A0A6J2FFJ4	*Zalophus californianus*

Structural-based features

In order to characterise the *KCNQ2* dataset using structural descriptors, it is necessary to know the structure of the protein in which each variant is found. However, the structure of the K_V7.2 channel is not fully solved as it only covers a fragment of its protein sequence (see Figure S2). This was a major challenge as a large number of the collected variants were located in areas of unknown structure. The following approaches were used.

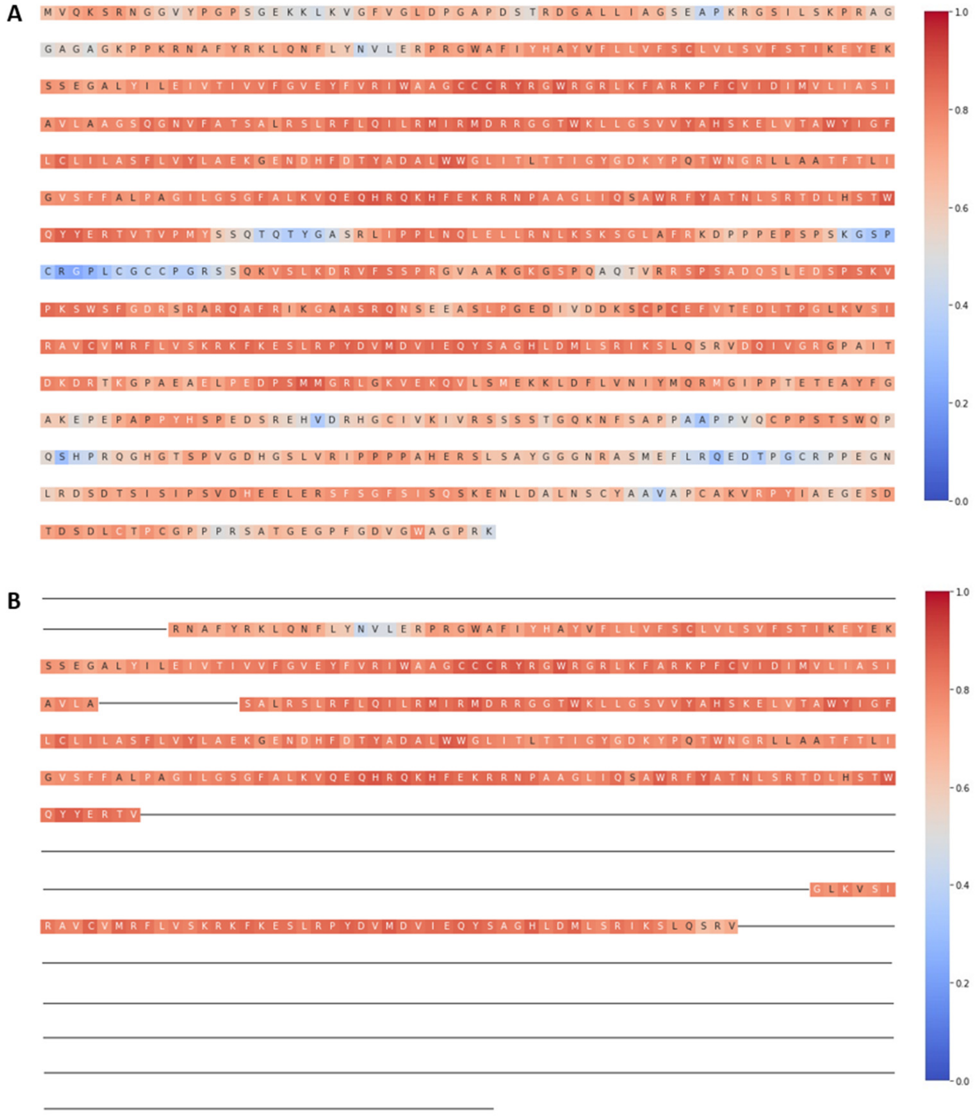

Figure S2. Complete sequence of the *KCNQ2* gene against the structurally solved sequence of the K_V7.2 channel. (A) Complete sequence of *KCNQ2* gene isoform 1 (UniProt ID: O43526) from residue 1 to 872. Each residue is coloured with its evolutive conservation value: red colour corresponding to more conserved residues (values close to 1) and blue colour to less conserved residues (values close to 0). **(B)** Structurally solved sequence of the K_V7.2 channel using 7CR3 PDB file as a reference. The residues whose structure is solved are coloured following the same pattern as in (A). The black lines simulate the sequence fragments of unknown structure.

1. <u>Location within the structural landscape of *KCNQ2* (*str_landscape*)</u>

It is a qualitative feature of structurally solved and unsolved regions of the K_v7.2 channel structure submitted in the RCSB Protein Data Bank (https://www.rcsb.org/) (PDB: 7CR3). We distinguish between GAPs and fragments (Figure S3).

<u>GAPs</u>

Correspond to regions (set of residues) with unsolved structure in the submitted 7CR3 file. In the specific case of the structure of the *KCNQ2* gene, 4 GAPs are identified:

- GAP 1 refers to amino acids located between positions 1-69.
- GAP 2 refers to amino acids located between positions 185-194.
- GAP 3 refers to amino acids located between positions 368-534.
- GAP 4 refers to amino acids located between positions 596-872.

<u>Fragments (FRAGs)</u>

Correspond to regions (set of residues) with solved structure in the submitted 7CR3 file. In the specific case of the structure of the *KCNQ2* gene, 3 fragments are identified:

- FRAG 1 refers to amino acids located between positions 70-184, which includes Cytoplasmic, Extracellular, S1, S2 and S3 topological domains.
- FRAG 2 refers to amino acids located between positions 195-367, which includes Cytoplasmic, Extracellular, S4, S5, S6, Pore, hA and hTW topological domains.
- FRAG 3 refers to amino acids located between positions 535-595, which includes Cytoplasmic, hB and hC topological domains.

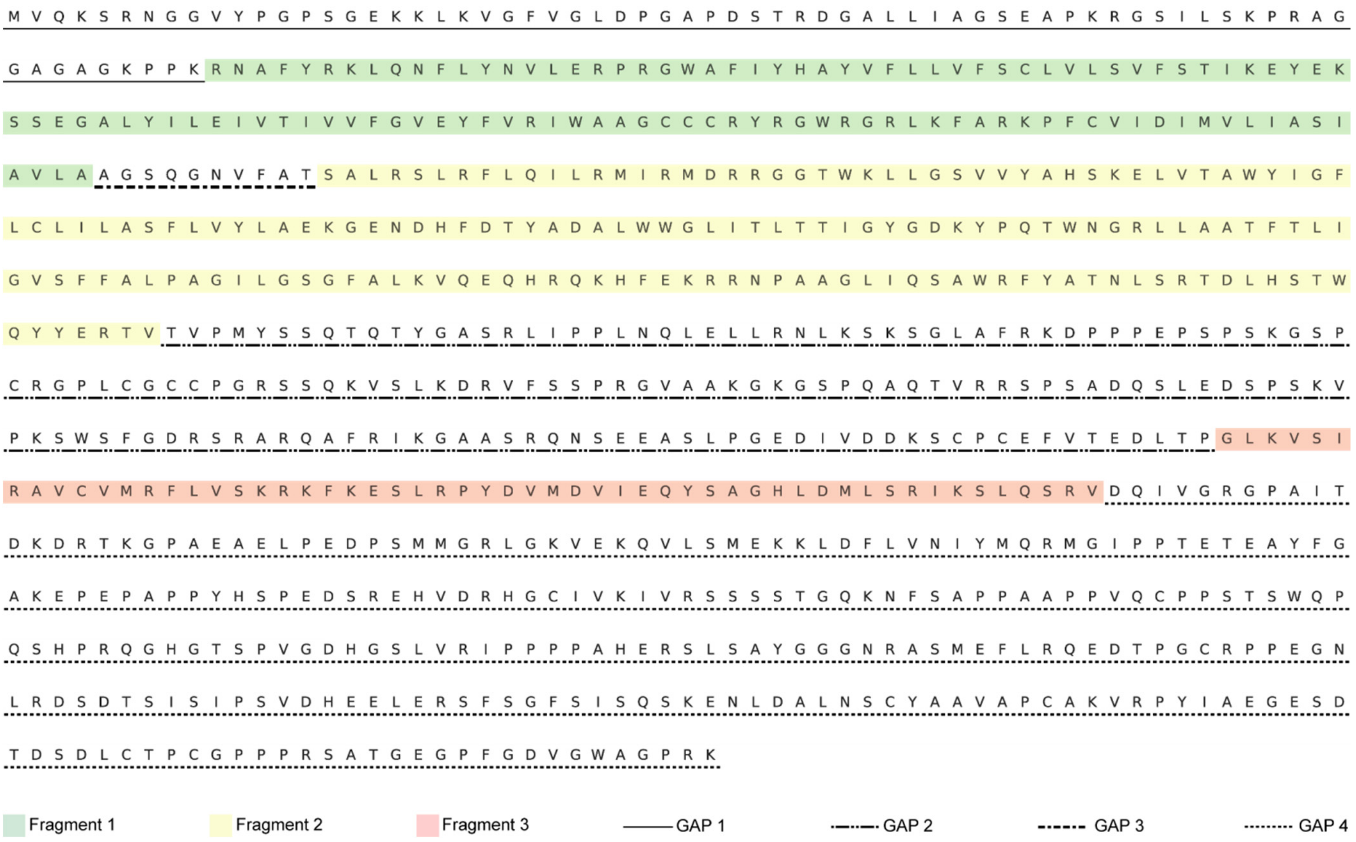

Figure S3. Structural landscape of the *KCNQ2* gene (isoform 1). The 4 GAPs correspond to regions with unsolved structure in the submitted 7CR3 PDB file, while the 3 fragments correspond to a set of residues already solved in the crystallographic structure.

2. <u>Secondary structure affected by the mutation *(secondary_str)*</u>

In order to understand the secondary structure affected by the mutation, it was necessary to combine the crystallographic structure of *KCNQ2* with secondary structure prediction softwares. Although there are several softwares for this purpose, the PROTEUS2 metaserver (http://www.proteus2.ca/proteus2/) (Montgomerie et al., 2008) was used. This software allows receiving the 7CR3 PDB file as input and modelling its unknown regions by homology modelling. The obtained results are shown in Figure S4.

```
  1  MVQKSRNGGVYPGPSGEKKLKVGFVGLDPGAPDSTRDGALLIAGSEAPKRGSILSKPRAG 60
     CCCCCCCCCCCCCHHHHHHHHHCCECCCCCCCCCCCCCCCCCCCCCCCCCCCCCCCCCCC
     998876677777765677777644557887677666666667778777776555777777

 61  GAGAGKPPKRNAFYRKLQNFLYNVLERPRGWAFIYHAYVFLLVFSCLVLSVFSTIKEYEK 120
     CCCCCCCCCCCCHHHHHHHHHHHHHHCCCCHHHTTTTTTTTTTTTTTTTTTTTTTCHHHH
     777777776664567887767767657876578999999999999999999999996777

121  SSEGALYILEIVTIVVFGVEYFVRIWAAGCCCRYRGWRGRLKFARKPFCVIDIMVLIASI 180
     HHHHHCTTTTTTTTTTTTTTTTTTTTTTCCCCCCCCCCCCCHHHHHHHCTTTTTTTTTTT
     666789999999999999999999999997887887666655467876599999999999

181  AVLAAGSQGNVFATSALRSLRFLQILRMIRMDRRGGTWKLLGSVVYAHSKELVTAWYIGF 240
     TTTTTTTCHHHHHHHHHHHHCTTTTTTTTTTTTTTTTCCHHHHHHHHHHHHCCCTTTTTT
     999999996665557889999999999999999999999999999999999999999999

241  LCLILASFLVYLAEKGENDHFDTYADALWWGLITLTTIGYGDKYPQTWNGRLLAATFTLI 300
     TTTTTTTTTTTTTTTTTTTCCCCCCCCTTTTTTTTTTTTTCCCCCCCCCCCTTTTTTTTT
     999999999999999999999999999999999999999999999999999999999999

301  GVSFFALPAGILGSGFALKVQEQHRQKHFEKRRNPAAGLIQSAWRFYATNLSRTDLHSTW 360
     TTTTTTTTTTCTTTTTTTTCHHHHHHHHHHHHHHHHHHHHHHHHHHHHHCCCCCCCHHHC
     999999999999999999999999999999999999999999998888657766544455

361  QYYERTVTVPMYSSQTQTYGASRLIPPLNQLELLRNLKSKSGLAFRKDPPPEPSPSKGSP 420
     CCCCCCCCCCCCCCCCCCCCCCCCCCCHHHHHCCCCCCCCCCCCCCCCCCCCCCCCCCCC
     676678887777777777777777645556557877777777777777777777777776

421  CRGPLCGCCPGRSSQKVSLKDRVFSSPRGVAAKGKGSPQAQTVRRSPSADQSLEDSPSKV 480
     CCCCCCCCCCCCCCCEECCCCCCCCCCCCCCCCCCCCCCCCCCCCCCCCCCCCCCCCCCC
     777776667667655557777677777777777777777777777777777777777777

481  PKSWSFGDRSRARQAFRIKGAASRQNSEEASLPGEDIVDDKSCPCEFVTEDLTPGLKVSI 540
     CCCCCCCCCCCCCCCCCCCCCCCCCCCCCCCCCCCCCCCCCCCCCCCCCCCCCCCCCCCE
     766777777777777777777777777777777766677666777776677777777543

541  RAVCVMRFLVSKRKFKESLRPYDVMDVIEQYSAGHLDMLSRIKSLQSRVDQIVGRGPAIT 600
     CHHHHHHHHHHHHHHHHHCCCCCHHHHHHHHHHCHHHHHHHHHHHHHHHHCCCCCCCCCC
     247999999999988888667666577889998645789999999988764456887777

601  DKDRTKGPAEAELPEDPSMMGRLGKVEKQVLSMEKKLDFLVNIYMQRMGIPPTETEAYFG 660
     CCCCCCCCCCCCCCCCCHHHHHHHHHHHHHHHHHHHHHHHHHHHHHHHCCCCCCCCCEEC
     777777777777766655788889999999999999999999888876587777654556

661  AKEPEPAPPYHSPEDSREHVDRHGCIVKIVRSSSSTGQKNFSAPPAAPPVQCPPSTSWQP 720
     CCCCCCCCCCCCCCCCCCCCCCCCCEEEEEEEEEECCCCCCCCCCCCCCCCCCCCCCCCC
     777777777777666666678887567887788856877666766777776777777777

721  QSHPRQGHGTSPVGDHGSLVRIPPPPAHERSLSAYGGGNRASMEFLRQEDTPGCRPPEGN 780
     CCCCCCCCCCCCCCCCCEEECCCCHHHHHHHHHHHHCCCCCCCCCCCCCCCCCCCCCCCC
     777777777777677765665676577766566547888887776766777777777777

781  LRDSDTSISIPSVDHEELERSFSGFSISQSKENLDALNSCYAAVAPCAKVRPYIAEGESD 840
     CCCCCCCCECCCCCCCCCCCCCCCCCCCCCCCCCCCCCCCCCCCCCCCCCCCCCCCCCCC
     777777655567765566677777776666666666777776667665667766678777

841  TDSDLCTPCGPPPRSATGEGPFGDVGWAGPRK 872
     CCCCCCCCCCCCCCCCCCCCCCCCCCCCCCCC
     77777777677766666777777766677778
```

Figure S4. Secondary structure prediction of K_v7.2 channel using PROTEUS2 and 7CR3 PDB file as a template. The first line refers to the *KCNQ2* protein sequence, the second one to the predicted secondary structure of which they are part (C = coiled coil, H = helix, T = transmembrane helix and E = beta strand) and the last one to the prediction score at each position, being 0 and 9 the lowest and highest value, respectively. A higher score value means more confidence in the prediction of the secondary structure of that position.

3. <u>AlphaFold2 pLDDT score *(plddt)*</u>

The prediction of the 3D structure of the $K_v7.2$ channel was performed using AlphaFold2 [Jumper *et al.,* 2021], the software of choice in this field. In addition to returning the 3D coordinates file, AlphaFold produces a per-residue estimate of its confidence on a scale from 0 - 100. This confidence measure is called pLDDT and corresponds to the model's predicted score on the lDDT-Cα metric, which is a superposition-free score that evaluates local distance differences of all atoms in a model, including validation of stereochemical plausibility (Mariani et al., 2013).

AlphaFold Protein Structure Database (https://alphafold.ebi.ac.uk/) (Varadi et al., 2021) provides guidance on the expected reliability of a given region, i.e., regions with pLDDT > 90 are expected to be modelled to high accuracy and regions with pLDDT between 70 and 90 are expected to be modelled well (a generally good backbone prediction). However, regions with pLDDT between 50 and 70 are low confidence and should be treated with caution and regions with pLDDT < 50 often have a ribbon-like appearance and should not be interpreted (probably suggesting that such region is either unstructured in physiological conditions or only structured as part of a complex).

The pLDDT score obtained with AlphaFold2 for each residue of the *KCNQ2* gene is provided in Data S4.

Reference List

1. (2022). UniProt: the Universal Protein Knowledgebase in 2023. Nucleic Acids Res.
2. Berman,H., Henrick,K., and Nakamura,H. (2003). Announcing the worldwide Protein Data Bank. Nat. Struct. Biol. *10*, 980.
3. Bogardt,R.A., Jr., Jones,B.N., Dwulet,F.E., Garner,W.H., Lehman,L.D., and Gurd,F.R. (1980). Evolution of the amino acid substitution in the mammalian myoglobin gene. J. Mol. Evol. *15*, 197-218.
4. Capra,J.A. and Singh,M. (2007). Predicting functionally important residues from sequence conservation. Bioinformatics. *23*, 1875-1882.
5. Jalalvand,E., Robertson,B., Tostivint,H., Wallen,P., and Grillner,S. (2016). The Spinal Cord Has an Intrinsic System for the Control of pH. Curr. Biol. *26*, 1346-1351.
6. Kyte,J. and Doolittle,R.F. (1982). A simple method for displaying the hydropathic character of a protein. J. Mol. Biol. *157*, 105-132.
7. Mariani,V., Biasini,M., Barbato,A., and Schwede,T. (2013). lDDT: a local superposition-free score for comparing protein structures and models using distance difference tests. Bioinformatics. *29*, 2722-2728.
8. Montgomerie,S., Cruz,J.A., Shrivastava,S., Arndt,D., Berjanskii,M., and Wishart,D.S. (2008). PROTEUS2: a web server for comprehensive protein structure prediction and structure-based annotation. Nucleic Acids Res. *36*, W202-W209.
9. Sievers,F. and Higgins,D.G. (2018). Clustal Omega for making accurate alignments of many protein sequences. Protein Sci. *27*, 135-145.
10. Varadi,M., Anyango,S., Deshpande,M., Nair,S., Natassia,C., Yordanova,G., Yuan,D., Stroe,O., Wood,G., Laydon,A., Zidek,A., Green,T., Tunyasuvunakool,K., Petersen,S., Jumper,J., Clancy,E., Green,R., Vora,A., Lutfi,M., Figurnov,M., Cowie,A., Hobbs,N., Kohli,P., Kleywegt,G., Birney,E., Hassabis,D., and Velankar,S. (2021). AlphaFold Protein Structure Database: massively expanding the structural coverage of protein-sequence space with high-accuracy models. Nucleic Acids Res.
